# 376. 7 versus 14 days of Antibiotic Treatment for Patients with Bloodstream Infections

**DOI:** 10.1093/ofid/ofae631.007

**Published:** 2025-01-29

**Authors:** Nick Daneman, Rob Fowler

**Affiliations:** Sunnybrook Health Sciences Centre, University of Toronto, Toronto, Ontario, Canada; Interdepartmental Division of Critical Care Medicine, Department of Medicine, Sunnybrook Health Sciences Centre, University of Toronto, Toronto, Ontario, Canada

## Abstract

**Background:**

Bloodstream infections are associated with substantial morbidity and mortality. Early, appropriate antibiotic therapy is important, but optimal duration of treatment is uncertain.

**Methods:**

In a multicenter, non-inferiority trial, we randomly assigned hospitalized (ward and intensive care unit [ICU]) patients with bloodstream infection to receive 7- versus 14-day antibiotic treatment.

Antibiotic selection, dosing and route were at the discretion of the treating team. We excluded patients with severe immune-suppression, foci requiring prolonged treatment, single cultures with potential contaminants, or cultures yielding *Staphylococcus aureus*. The primary outcome was 90-day mortality, with a 4% absolute non-inferiority margin.

**Results:**

Across 74 hospitals in 7 countries, 3608 patients were randomized and included in the intention-to-treat analysis (1,814 to 7-day and 1,794 to 14-day antibiotic treatment). At enrolment, 55% of patients were in the ICU and 45% on hospital wards. Infections were community-onset (75.4%), hospital-acquired (13.4%) and ICU-acquired (11.2%). Bacteremia sources were most commonly from the urinary tract (42.2%), abdomen (18.8%), lung (13.0%), vascular catheters (6.3%), and skin or soft tissue (5.2%). The primary outcome of 90-day mortality occurred in 261 (14.5%) patients receiving 7-day and in 286 (16.1%) receiving 14-day treatment (absolute difference -1.6% [95.7% confidence interval -4.0 to 0.8]), demonstrating non-inferiority of shorter duration treatment. A per-protocol analysis also demonstrated non-inferiority (-2.0% [95% confidence interval -4.5 to 0.6]). These findings were generally consistent across secondary clinical outcomes, and pre-specified patient, pathogen and syndrome subgroups.

**Conclusion:**

Treating hospitalized patients with bloodstream infection with antibiotics for 7 days is non-inferior to treating for 14 days.

(Funded by the Canadian Institutes of Health Research, Health Research Council of New Zealand, Australian National Medical Research Council, Physicians Services Incorporated Ontario and Ontario Ministry of Health and Long-term Care Innovation Fund; ClinicalTrials.gov NCT03005145)

**Disclosures:**

**All Authors**: No reported disclosures

